# Importance of Vaccination against SARS-CoV-2 in Patients with Interstitial Lung Disease Associated with Systemic Autoimmune Disease

**DOI:** 10.3390/jcm11092437

**Published:** 2022-04-26

**Authors:** Natalia Mena-Vázquez, Aimara García-Studer, Marta Rojas-Gimenez, Carmen María Romero-Barco, Sara Manrique-Arija, Arkaitz Mucientes, María Luisa Velloso-Feijoo, Francisco Javier Godoy-Navarrete, Pilar Morales-Garrido, Rocío Redondo-Rodríguez, MC Ordoñez-Cañizares, Rafaela Ortega-Castro, Jose Manuel Lisbona-Montañez, Ana Hidalgo Conde, Rocío Arnedo Díez de los Ríos, Eva Cabrera César, Francisco Espildora, María Carmen Aguilar-Hurtado, Isabel Añón-Oñate, Inmaculada Ureña-Garnica, Antonio Fernández-Nebro

**Affiliations:** 1 Instituto de Investigación Biomédica de Málaga (IBIMA), 29010 Malaga, Spain; aimara.garcia95@gmail.com (A.G.-S.); menchu01@hotmail.com (C.M.R.-B.); sarama_82@hotmail.com (S.M.-A.); arkaitzmucientes@gmail.com (A.M.); rocioredondo91@hotmail.com (R.R.-R.); maricarmenoc@hotmail.com (M.O.-C.); inuregar@gmail.com (I.U.-G.); afnebro@gmail.com (A.F.-N.); 2 UGC de Reumatología, Hospital Regional Universitario de Málaga, 29009 Malaga, Spain; josemanuellisbona@hotmail.com; 3 Instituto Maimónides de Investigación Biomédica de Córdoba (IMIBIC), 14004 Cordoba, Spain; rojasgimenezm@gmail.com (M.R.-G.); orcam84@hotmail.com (R.O.-C.); 4 UGC de Reumatología, Hospital Universitario Reina Sofía de Córdoba, 14004 Cordoba, Spain; 5 UGC de Reumatología, Hospital Clínico Universitario Virgen de la Victoria, 29010 Malaga, Spain; 6 Departamento de Medicina, Universidad de Málaga, 29016 Malaga, Spain; 7 UGC de Reumatología, Hospital Universitario Virgen de Valme, 41014 Sevilla, Spain; mlvelloso@hotmail.com; 8 Hospital Universitario de Jaén, 23007 Jaén, Spain; fjgodoynavarrete@gmail.com (F.J.G.-N.); isaanononate@gmail.com (I.A.-O.); 9 Hospital Universitario San Cecilio, 18200 Granada, Spain; pimoraga@gmail.com; 10 Servicio de Medicina Interna, Hospital Universitario Virgen de la Victoria, 29010 Malaga, Spain; ahidalgoconde@gmail.com (A.H.C.); rocioardiez@gmail.com (R.A.D.d.l.R.); 11 UGC Neumología, Hospital Universitario Virgen de la Victoria, 29010 Malaga, Spain; evacabreracesar@gmail.com; 12 UGC de Neumología, Hospital Regional Universitario de Málaga, 29009 Malaga, Spain; fespildorahernandez@gmail.com; 13 UGC de Radiodiagnóstico, Hospital Regional Universitario de Málaga, 29009 Malaga, Spain; maguh007@gmail.com

**Keywords:** interstitial lung disease (ILD), systemic autoimmune disease (SAD), SARS-CoV-2 infections, vaccination COVID-19

## Abstract

Objectives: To describe the frequency of COVID-19 and the effect of vaccination in patients with interstitial lung disease and systemic autoimmune disease (ILD-SAD) and to identify factors associated with infection and severity of COVID-19. Methods: We performed a cross-sectional multicenter study of patients with ILD-SAD followed between June and October 2021. The main variable was COVID-19 infection confirmed by a positive polymerase chain reaction (PCR) result for SARS-CoV-2. The secondary variables included severity of COVID-19, if the patient had to be admitted to hospital or died of the disease, and vaccination status. Other variables included clinical and treatment characteristics, pulmonary function and high-resolution computed tomography. Two logistic regression was performed to explore factors associated with “COVID-19” and “severe COVID-19”. Results: We included 176 patients with ILD-SAD: 105 (59.7%) had rheumatoid arthritis, 49 (27.8%) systemic sclerosis, and 22 (12.54%) inflammatory myopathies. We recorded 22/179 (12.5%) SARS-CoV-2 infections, 7/22 (31.8%) of them were severe and 3/22 (13.22%) died. As to the vaccination, 163/176 (92.6%) patients received the complete doses. The factors associated with SARS-CoV-2 infection were FVC (OR (95% CI), 0.971 (0.946–0.989); *p* = 0.040), vaccination (OR (95% CI), 0.169 (0.030–0.570); *p* = 0.004), and rituximab (OR (95% CI), 3.490 (1.129–6.100); *p* = 0.029). The factors associated with severe COVID-19 were the protective effect of the vaccine (OR (95% CI), 0.024 (0.004–0.170); *p* < 0.001) and diabetes mellitus (OR (95% CI), 4.923 (1.508–19.097); *p* = 0.018). Conclusions: Around 13% of patients with ILD-SAD had SARS-CoV-2 infection, which was severe in approximately one-third. Most patients with severe infection were not fully vaccinated.

## 1. Introduction

Diffuse interstitial lung disease (ILD) is a frequent complication of systemic autoimmune diseases (SADs) and entails high morbidity and mortality [1]. The SADs most commonly associated with ILD (ILD-SAD) include systemic sclerosis (SSc), rheumatoid arthritis (RA), and inflammatory myopathy (IM), which have been reported in 70% of affected patients [2]. Patients with ILD-SAD have a greater risk of infections stemming from the disease itself and its treatments, thus further increasing morbidity and mortality [3].

Coronavirus 2019 disease (COVID-19), which is caused by SARS-CoV-2, has proven challenging for health care throughout the world. The course of COVID-19 varies considerably, ranging from absence of symptoms or only mild flu-like symptoms to very severe disease with acute respiratory syndrome, multi-organ involvement, and death [4]. Part of this clinical variability is due to the patient’s own characteristics. Since the start of the pandemic, several comorbid conditions and risk factors have been identified. These are associated with a greater risk of infection and severity of COVID-19 and include older age, male sex, cardiovascular risk factors (i.e., obesity, arterial hypertension, diabetes mellitus), immunosuppression, and chronic diseases affecting the lungs, liver, and kidneys [5,6,7,8]. Independently of their immunosuppressive treatment, patients with SADs have a greater risk of developing severe COVID-19 [4,9].

In addition to lung involvement, patients with ILD-SAD are usually affected by immunosuppression, which leaves them more likely to develop severe COVID-19 than the rest of the population. Furthermore, this immunosuppression could reduce their response to vaccines. However, evidence on COVID-19 and vaccination in patients with ILD-SAD is very limited [10,11,12]. Lee et al. [11] recently studied a cohort of 8070 patients with COVID-19, of whom 67 previously had ILD, both in association with SADs and secondary to other conditions or as an idiopathic presentation, and found that patients with ILD were at greater risk of severe COVID-19 than the rest of the population (adjusted OR, 2.23; 95% CI 1.24–4.01). A further three studies have reported similar results [13,14,15], stating that patients with ILD are at greater risk of severe COVID-19 and death.

Few studies have evaluated the association between COVID-19 and ILD-SAD. If we are to identify the most vulnerable patients, then we must determine the risk factors associated with more severe COVID-19, as well as the effect of vaccines in this population. Therefore, the objectives of our study were as follows: (1) to describe the frequency and severity of COVID-19 and the effect of vaccination; and (2) to identify risk factors associated with infection and severity of COVID-19 in patients with ILD-SAD and COVID-19 in various university hospitals in Andalusia, Spain.

## 2. Materials and Methods

### 2.1. Design

We performed a cross-sectional multicenter cohort study of patients with ILD-SAD who were prospectively followed at 7 university hospitals in Andalusia, Spain. Recruitment was between June and October 2021. The study was approved by the Research Ethics Committee of Hospital Regional Universitario de Málaga, Málaga, Spain (Code 0868-N-20).

### 2.2. Study Population

We included all patients with ILD-SAD in follow-up in rheumatology clinics at different centers throughout Andalusia between June and October 2021. ILD was confirmed using pulmonary function tests (PFTs) and high-resolution computed tomography (HRCT) or lung biopsy. The inclusion criteria were as follows: age ≥18 years, RA classified according to the criteria of the 2010 American College of Rheumatology (ACR)/European League Against Rheumatism (EULAR) [16], SSc classified according to the criteria of ACR/EULAR 2013 [17], or dermato-myositis and poly-myositis (IM) classified according to the criteria of Bohan and Peter [18,19], as applicable. Patients with any inflammatory or rheumatic disease other than RA, SSc, or IM were excluded (except secondary Sjögren syndrome).

### 2.3. Protocol

All patients were evaluated based on a pre-established protocol for collecting clinical disease-related data every 3 to 6 months at the rheumatology clinic. Data on COVID-19 infection and vaccination were recorded in the electronic clinical history and confirmed through the official vaccination registry, which could be accessed from the patient’s clinical history. All patients underwent HRCT and PFT in 2020 in order to evaluate progression of lung disease [20], before COVID19 infection. All HRCT scans were based on axial slices (1.5 or 2.0 mm in thickness) taken at 1-cm intervals along the thorax and reconstructed using a high-spatial-frequency algorithm, with acquisition of 20–25 images per patient.

### 2.4. Variable and Case Definitions

The main variable was COVID-19 infection confirmed by a positive polymerase chain reaction (PCR) result for SARS-CoV-2 from the start of the pandemic, in March 2020, until October 2021. Recruitment was between June and October 2021. The secondary variables included severity of COVID-19 and vaccination status. COVID-19 was defined as severe if the patient had to be admitted to hospital or died of the disease. A patient was considered to have been admitted to hospital if he/she was admitted to the observation area or hospitalization area, including the intensive care unit, for at least 24 h [21]. Following the guidelines of the technical working group for COVID-19 vaccination of October 2021 in Andalusia, Spain, the patients included in our study were in one of two defined groups: patients >65 years or ≤65 years with high-risk conditions. For these groups of patients, it was considered correctly vaccinated if he/she had received the full vaccine schedule of at least 2 doses. The time frame in which these patients were inoculated with mRNA vaccines (Pfizer or Moderna) was different with the Pfizer vaccine (3 weeks) than with the Moderna one (4 weeks). The vaccination schedule is shown in Supplementary Figure S1 [22]. We also recorded the interval between infection and vaccination.

### 2.5. Other Variables

Other variables included the following: epidemiological characteristics such as sex (male/female) and age (years) and cardiovascular risk factors such as arterial hypertension, diabetes mellitus and obesity. Arterial hypertension was defined as an arterial pressure ≥140/90 mmHg or current treatment with antihypertensive medication. Diabetes mellitus were diagnosed based on the recommendations of the American Diabetes Association 2010 and obesity with body mass index ≥30 [5,6,7,8]; clinical-laboratory characteristics such as time since diagnosis, diagnostic delay, and smoking history (current or previous). We also collected a series of laboratory variables, as follows: autoantibodies; rheumatoid factor (RF) (reference value, 20 U/mL; high titers >60 U/mL); anti-citrullinated peptide antibodies (ACPA) (reference value, 10 U/mL, high values >340 U/mL); antinuclear antibodies (ANA); anti-U1RNP antibodies (MCTD); anti-Scl70, anti-RNA polymerase III, and anti-PM-Scl (PM-Scl overlap); anti-Ro 52 kDa, anti-Ro 60 kDa, anti-La, anti-aminoacyl-tRNA synthetase, anti-Mi-2, anti-SRP, anti-TIF1, anti-NXP-2/MJ, anti-MDA5 (CADM), anti-HMGCR, and anti-SAE. We recorded all treatments with conventional synthetic disease-modifying anti-rheumatic drugs (csDMARDs) that are the traditional drugs: methotrexate, leflunomide, sulfasalazine or hydroxy-chloroquine; targeted synthetic DMARDs (tsDMARDs) that consist of synthetic small molecules targeting intracellular transduction pathways: Janus kinase (JAK) inhibitors (tofacitinib and baricitinib); biologic DMARDS (bDMARDs) that are produced using molecular biology (recombinant DNA) techniques such as tumor necrosis factor inhibitors or biologics that target other molecules like CD80/CD86 ligands expressed in presenting cells that mediates the pathway in T-cell co-stimulation (abatacept), the interleukin-6 inhibitor and agents that eliminates CD20-positive B cells (rituximab). We have also collected data of treatment with anti-fibrotics (nintedanib), corticosteroids and others immuno-suppressants (myco-phenolate mofetil, cyclophosphamide, and azathioprine).

As for data on ILD, we evaluated the radiological pattern based on the most recent HRCT scan according to the criteria of the American Thoracic Society/European Respiratory Society International Multidisciplinary Consensus Classification of the Idiopathic Interstitial Pneumonias [23] and classified it into 3 patterns: nonspecific interstitial pneumonia (NSIP), usual interstitial pneumonia (UIP), and other (bronchiolitis obliterans, organizing pneumonia, lymphoid interstitial pneumonia, and mixed). PFTs included complete spirometry expressed as percent predicted and adjusted for age, sex, and height. The diffusing capacity of the lung for carbon monoxide (DLCO) was evaluated using the single-breath method (DLCO-SB). We recorded lung function before COVID19 infection.

### 2.6. Statistical Analysis

We performed a descriptive analysis of the main characteristics of all the subgroups of patients with ILD-SAD using the χ^2^ and ANOVA or Kruskal-Wallis test (depending on the normality of the distribution). Comparisons of the main characteristics between patients with ILD-SAD with and without COVID-19 and between patients with severe COVID-19 or not were performed using the χ^2^ and *t* test or Mann-Whitney test. Qualitative variables are expressed as whole numbers and percentages and quantitative variables as mean (SD) or median (IQR) depending on the distribution. Normality was confirmed using the Kolmogorov-Smirnov test. Finally, we ran 3 stepwise logistic regression models to explore those variables that were independently associated with “COVID-19” and “severe COVID-19” in patients with ILD-SAD. Variables that proved to be statistically significant in the bivariate analysis or were of clinical interest were included in the model. Statistical significance was set at *p* < 0.05 for all the analyses. All data were analyzed using R 2.4-0.

## 3. Results

### 3.1. Baseline Characteristics of Patients with ILD-SAD

The study population comprised 176 patients with ILD-SAD, of whom 105 (59.7%) had RA, 49 (27.8%) SSc, and 22 (12.54%) IM. Their clinical, epidemiological, and therapy-related characteristics at inclusion are shown in Table 1. More than half of the patients were women (66.5%); the mean age was 64 years, and the median (IQR) time since diagnosis of ILD was 56.5 months (28.7–96.7). All the patients were taking treatment for ILD at inclusion; most were taking a csDMARD (63%) and almost 40% were taking a bDMARD or immunosuppressant. The most frequent bDMARD was rituximab (32/176 patients [18.2%]).

The 3 groups of patients differed with respect to many epidemiological, clinical, laboratory, and therapy-related characteristics. Compared with the other groups, patients with RA were more frequently male, with a higher mean age, and more frequently smokers. Similarly, RF and ACPA were more frequent findings in patients with RA (90%); the most common antibody in SSc was anti-Scl70 (61%), and the most common antibody in IM was anti-Jo-1 (36%). The different autoantibodies profiles in different patients with ILD-SAD are shown in Supplementary Table S1. As for treatment, patients with RA more frequently took csDMARDs (*p* < 0.001), whereas immuno-suppressants were more common among patients with SSc and IM (*p* < 0.001).

The most common radiologic pattern was NSIP, in 91/176 patients (51.7%), followed by UIP, in 75/176 patients (42.6%) and fibrotic NSIP in 10/176 patients (5.7%). By subgroup, NSIP was the most common pattern in patients with IM (90.9%) and SSc (79.6%), and UIP was the most common pattern in RA (62.9%). Lung function before COVID19 infection is shown in Table 1.

### 3.2. Characteristics of COVID-19 Infection and Vaccination in Patients with ILD-SAD

Twenty-two patients (12.5%) had SARS-CoV-2 infection; infection was severe in seven patients (31.8% of the infected and 3.9% of the complete cohort), and three patients (13.6% of the infected and 1.67% of the complete cohort) died. In all patients, the COVID-19 infection was after the HRCT was performed. Only four cases of COVID infection were in 2020 (two patients in October, one in November and one in December) and after the HRCT was performed. The rest of the cases of COVID infection occurred during 2021. There were no significant differences between the subgroups of patients with respect to SARS-CoV-2 infection, although compared with the other groups, more patients with RA had to be admitted to hospital or died. These differences were not statistically significant (Table 2). After 3–6 months of COVID-19 infection, the patients had no flares of their autoimmune conditions. However, after 3–6 months of follow-up in the 22/176 patients with SARS-CoV-2 infection compared to before COVID19 infection, there was impairment of FVC (67.8 (15.0) vs. 72.0 (19.9); *p* = 0.043), DLCO (51.2 (14.8) vs. 55.7 (16.3); *p* = 0.039), and FEV1 (69.4 (15.8) vs. 73.5 (18.6); *p* = 0.093).

A total of 163/176 patients (92.6%) were fully vaccinated with mRNA against COVID-19 (Pfizer-BioNTech or Moderna). Of these 163 patients, 18 (11%) were infected with SARS-CoV-2. Four of the 18 patients (22.2%) became infected after vaccination, and 14 (77.7%) became infected before they had been fully vaccinated. Of the 13 patients who were not vaccinated, four (30.7%) had severe COVID-19 (three died). The patients had no flares of their autoimmune conditions after vaccination. A total of 24/163 patients (14.7%) described general malaise and self-limited low-grade fever 48 h after vaccination. All the non-vaccinated patients chose not to be vaccinated owing to a fear of the adverse effects of the vaccine.

### 3.3. Factors Associated with COVID-19 in Patients with ILD-SAD

Compared with uninfected patients, the median (IQR) time to onset of ILD in the 22/176 patients with SARS-CoV-2 infection (12.5%) was greater (80.9 (48.5–114.1) vs. 59.1 (28.5–97.1); *p* = 0.041), as was impairment of FVC (63.9 (18.5) vs. 74.9 (19.8); *p* = 0.015), DLCO (51.9 (14.3) vs. 56.1 (16.5); *p* = 0.045), and FEV_1_ (65.2 (17.4) vs. 76.8 (18.4); *p* = 0.030). As for treatment, patients with SARS-CoV-2 infection more frequently took rituximab (7 (31.8) vs. 25 (16.0); *p* = 0.040) and glucocorticoids (18 (81.8) vs. 97 (63.0); *p* = 0.043); in addition, the median (IQR) dose of glucocorticoids was greater (5.0 (5.0–10.0) vs. 5.0 (0.0–6.0); *p* = 0.034). Patients not infected by SARS-CoV-2 were more frequently vaccinated (146 (94.8) vs. 17 (77.3); *p* = 0.003) (Supplementary Table S2).

Table 3 shows the results of the multivariate logistic regression analysis for the dependent variable SARS-CoV-2 infection in patients with ILD-SAD. The variables that were independently associated with SARS-CoV-2 infection were FVC (OR (95% CI), 0.971 (0.946–0.989); *p* = 0.040), vaccination (OR (95% CI), 0.169 (0.030–0.570); *p* = 0.004), and treatment with rituximab (OR (95% CI), 3.490 (1.129–6.100); *p* = 0.029).

### 3.4. Factors Associated with Severity of COVID-19 Infection in Patients with ILD-SAD

Seven patients with ILD-SAD (3.9%) had severe infection requiring admission to hospital, three died (1.7%). As for the radiological pattern of patients who had severe infection, four/seven patients (51.1%) had UIP pattern, two/seven patients (28.6) had NSIP pattern, and one patient (14.3%) had fibrotic NSIP pattern. Compared with patients who did not have severe SARS-CoV-2 infection, those who had severe infection had previously experienced greater impairment of FVC (62.5 (23.4) vs. 75.9 (19.7); *p* = 0.022), DLCO (50.9 (18.1) vs. 55.7 (16.3); *p* = 0.049), and FEV_1_ (64.9 (12.3) vs. 74.1 (19.7); *p* = 0.043). In addition, they had more frequently received rituximab (3 (42.9) vs. 29 (17.2); *p* = 0.048) and glucocorticoids (7 (100.0) vs. 108 (63.9); *p* = 0.036), with a higher median (IQR) of glucocorticoids (mg/day) (6.2 (5.0–11.2) vs. 5.0 (0.0–7.5); *p* = 0.039), and included a greater number of diabetes mellitus patients (4 (57.1) vs. 14 (8.3); *p* = 0.001) and a lower number of vaccinated patients (2 (28.6) vs. 161 (95.3); *p* < 0.001) (Supplementary Table S3). The radiologic subtype of ILD was not associated with infection or severe infection (Supplementary Tables S2 and S3).” Table 4 shows the results of a multivariate logistic regression analysis for the dependent variable severe SARS-CoV-2 infection in patients with ILD-SAD. The factors associated with severe COVID-19 were the protective effect of the vaccine (OR (95% CI), 0.024 (0.004–0.170); *p* < 0.001) and diabetes mellitus (OR (95% CI), 4.923 (1.508–19.097); *p* = 0.018).

On the other hand, the characteristics of patients with severe SARS-CoV2 infection and SARS-CoV2 infection are shown in Supplementary Table S4. An alternative multivariate analysis was conducted to study factors associated with severe SARS-CoV2 infection comparison of severe and non-severe COVID. The factors associated with severe COVID-19 in IDL-SAD patients with SARS-CoV2 infection was the protective effect of the vaccine (OR (95% CI), 0.020 (0.002–0.179); *p* < 0.001) (Table 5).

## 4. Discussion

We attempted to describe the frequency of, and factors associated with, the severity of COVID-19, as well as the effect of vaccination against SARS-CoV-2 in patients with ILD-SAD. We observed that around 13% of affected patients with ILD-SAD had PCR-confirmed SARS-CoV-2 infection and that, of these, around one-third had severe infection. The frequency of COVID-19 we recorded was somewhat lower than that reported elsewhere for patients with ILD, probably because our study only included patients with SAD-type ILD, whereas other studies address other lung diseases and ILD of various etiologies. For example, in their large registry of patients with COVID-19, Jaime Signes-Costa et al. [24] found that around 22% had previously had lung disease, mainly COPD or asthma, and that 8.1% had ILD of various causes. Similarly, Drake et al. [14] found that 161 of 349 patients (46%) with ILD had PCR-confirmed COVID-19. However, most of these patients (42.2%) had ILD associated with idiopathic pulmonary fibrosis or other types, whereas only 14.3% had ILD associated with SAD. Furthermore, in our study, up to one-third of patients with ILD-SAD had severe infection; this finding is in line with those of other studies showing that around 30% of patients with ILD of various causes and COVID-19 required admission to hospital or died [24,25]. Several studies have also shown that, in patients with COVID-19 and ILD, the disease manifested more severely than in those who did not have ILD [11,13,26].

One of the factors that was independently associated with SARS-CoV-2 infection in patients with ILD-SAD in our study was treatment with rituximab. Similarly, two studies that evaluate the risk of infection by COVID-19 in persons with SAD found that rituximab could be associated with a greater risk of being infected by SARS-CoV-2 [27,28]. Furthermore, a recent systematic review and meta-analysis that evaluated 90 studies [29] reported that patients who receive anti-CD20 treatment, even those who were recently treated (<6 months after rituximab) or who had depleted B-cell counts, are at a high risk of not seroconverting and, therefore, becoming infected by SARS-CoV-2 despite developing humoral immune responses and responses mediated by other cell types after vaccination.

Other factors that protected against infection included greater FVC and vaccination. Studies have shown that more marked impairment of FVC, as well as more extensive lung fibrosis in patients with ILD before COVID-19 infection, increases the risk and severity of infection [30,31]. Nevertheless, this has been evaluated mainly in ILD associated with idiopathic pulmonary fibrosis, since, to date, no studies have included only patients with ILD-SAD. Most patients in our study were fully vaccinated, although 13 chose not to be vaccinated. Vaccination against SARS-CoV-2 was associated with a lower risk of infection and lower risk of severe infection. Although some initial clinical trials performed with SARS-CoV-2 vaccine excluded patients with SAD [32,33], a subsequent systematic review of several observational studies showed that sero-conversion rates after a full course of mRNA vaccination were very good (>90%) in patients with SAD who did not receive most of the treatments used in this group, although the number of patients who received anti-CD20 or CTLA4 therapy could be lower [34]. Until we have more data on the effect of the vaccine in immunosuppressed patients with ILD-SAD, it remains important to minimize the possibility of SARS-CoV-2 infection and severe COVID-19 infection in affected patients, who should be prioritized for vaccination [35]. Another factor associated with severe COVID-19 was diabetes mellitus. In this sense, a meta-analysis observed that the presence of diabetes was associated with a significantly increased risk of mortality in patients admitted to the hospital with COVID-19 [36]. Therefore, in patients with ILD-SAD, we should consider cardiovascular risk factors such as diabetes mellitus, which have been associated with a worse prognosis in the general population.

Our study is subject to a series of limitations. First, the sample of patients with ILD-SAD is small and there is no control group; therefore, our results should be interpreted with caution. Nevertheless, we did identify factors associated with SARS-CoV-2 infection and severe COVID-19 in this population. Furthermore, our data support the recommendations of international scientific societies on vaccination in patients with SAD, where the risk of infection was greater in those with more severe disease [37]. We observed a greater risk of SARS-CoV-2 infection in ILD-SAD patients treated with RTX. Previous studies have shown altered COVID19 vaccine sero-conversion with immuno-modulating drugs such as RTX and, therefore, despite vaccination, subjects who received immunotherapy have shown susceptibility to SARS-CoV-2 infection [27,28,29]. Unfortunately, SARS-CoV-2 serologic status of the patients has not been included, as in our center the determination of SARS-CoV-2 antibodies is not a routine practice. Another limitation is the retrospective design of the study. Our patients probably suffered infections of different SARS-CoV-2 variants and received variable COVID19 management depending on the momentum of the pandemic. Nevertheless, our primary variable, severe COVID 19, was not influenced by this, as defined as a disease that resulted in hospitalization or mortality. Furthermore, given that we only included patients with PCR-confirmed SARS-CoV-2, some patients with COVID-19 could have been excluded. In addition, at the beginning of the pandemic, the SARS-CoV-2 PCR test was not performed on all patients. However, we did confirm that the remaining patients did not have respiratory symptoms or, if they did, we confirmed that their PCR result was negative. Furthermore, although we considered various SADs with different pathogenic mechanisms that could affect SARS-CoV-2 infection differently, we were able to provide data on infection and vaccination for each type of disease. In relation to the timeframe of vaccination, following the guidelines of the technical working group for COVID-19 vaccination of October 2021 in Andalusia, Spain, we consider as correctly vaccinated those who had received the full vaccine schedule of at least two doses. Although in these guidelines the administration of a third dose of vaccine began to be referred to, the last patient recruited was in October 2021, so there was no time to collect the data for the third vaccine and we consider as complete vaccination two doses of the vaccine. Finally, although we considered various SADs with different pathogenic mechanisms that could affect SARS-CoV-2 infection differently, we were able to provide data on infection and vaccination for each type of disease. Finally, despite the cross-sectional design of our analysis, clinical, laboratory, HRCT, and PFT data were available for all patients during the study period, since they had previously been requested for analysis of patients’ progress [20].

## 5. Conclusions

In conclusion, around 13% of patients with ILD-SAD had SARS-CoV-2 infection, which was severe in approximately one-third. Most patients with severe infection were not fully vaccinated. A more marked impairment of FVC, and treatment with rituximab were associated with a greater risk of COVID-19, whereas vaccination was a protective factor for both mild and severe infection.

## Figures and Tables

**Table 1:** Clinical-epidemiological characteristics of 176 patients with ILD-SAD.

Variable	Total (*n* = 176)	Rheumatoid Arthritis *n* = 105	Systemic Sclerosis *n* = 49	Inflammatory Myopathy *n* = 22	*p*-Value
Baseline clinical-epidemiological characteristics					
Female sex, *n* (%)	117 (66.5)	58 (55.2)	42 (85.7)	17 (77.3)	<0.001
Age in years, mean (SD)	64.4 (12.7)	67.9 (9.6)	60.9 (12.5)	55.7 (18.9)	<0.001
Smoking history					0.116
Never smoked, *n* (%)	120 (68.2)	67 (63.8)	34 (69.4)	19 (86.4)	
Smoked (%)	56 (31.8)	38 (36.2)	15 (30.6)	3 (13.6)	
Arterial hypertension, *n* (%)	52 (29.5)	34 (32.4)	10 (20.4)	8 (36.4)	0.239
Diabetes mellitus, *n* (%)	18 (10.2)	14 (13.3)	1 (4.5)	3 (6.1)	0.250
Obesity (BMI ≥ 30), *n* (%)	36 (20.5)	26 (24.8)	7 (14.3)	3 (13.6)	0.226
Time since diagnosis of SAD, months, mean (IQR)	139.0 (57.4–217.7)	150.8 (63.9–238.4)	143.4 (67.5–229.6)	61.6 (45.1–159.1)	0.035
Time since diagnosis of ILD, months, median (IQR)	56.5 (28.7–96.7)	46.2 (25.4–83.0)	67.3 (52.5–87.6)	30.9 (25.1–72.5)	0.071
Radiological pattern					<0.001
NSIP, *n* (%)	91 (51.7)	32 (30.5)	39 (79.6)	20 (90.9)	
UIP, *n* (%)	75 (42.6)	66 (62.9))	8 (16.3)	1 (4.5) (90.9)	
f-NSIP, *n* (%), *n* (%)	10 (5.7)	7 (6.7)	2 (4.1)	1 (4.5)	
PFT results					
FVC% predicted, mean (SD)	72.0 (19.9)	70.7 (19.9)	71.3 (21.4)	73.1 (16.6)	0.471
FEV1% predicted, mean (SD)	73.5 (18.6)	71.2 (19.3)	72.2 (17.3)	74.4 (17.9)	0.345
DLCO-SB% predicted, mean (SD)	55.7 (16.3)	54.3 (16.5)	52.4 (15.9)	60.8 (15.2)	0.140
Treatments					
csDMARDs, *n* (%)	111 (63.1)	90 (85.7)	10 (18.2)	11 (50.0)	<0.001
Methotrexate, *n* (%)	53 (30.6)	45 (44.1)	4 (8.2)	4 (18.2)	<0.001
Leflunomide, *n* (%)	29 (16.8)	28 (27.5)	1 (2.0)	0 (0.0)	<0.001
Sulfasalazine, *n* (%)	8 (4.5)	7 (6.7)	0 (0.0)	1 (4.5)	<0.001
Hydroxy-chloroquine, *n* (%)	30 (17.0)	20 (19.0)	5 (10.2)	5 (22.7)	0.313
bDMARDs, *n* (%)	67 (38.7)	47 (46.1)	14 (28.6)	6 (27.3)	0.059
Anti-TNF, *n* (%)	8 (4.6)	8 (7.7)	0 (0.0)	0 (0.0)	0.018
Tocilizumab, *n* (%)	7 (4.0)	4 (3.8)	1 (2.0)	2 (9.1)	0.369
Abatacept, *n* (%)	20 (11.4)	20 (19.0)	0 (0.0)	0 (0.0)	<0.001
Rituximab, *n* (%)	32 (18.2)	15 (14.3)	13 (26.5)	4 (18.2)	0.186
Immuno-suppressants, *n* (%)	65 (36.9)	16 (15.2)	32 (65.3)	17 (77.3)	<0.001
Mycophenolate, *n* (%)	48 (27.3)	10 (9.5)	26 (53.1)	12 (54.5)	<0.001
Azathioprine, *n* (%)	14 (8.0)	5 (4.8)	4 (8.2)	5 (22.7)	<0.001
Cyclophosphamide, *n* (%)	3 (1.7)	0 (0.0)	2 (4.1)	1 (4.5)	0.111
Anti-fibrotic, *n* (%)	3 (1.7)	2 (2.0)	1 (2.0)	0 (0.0)	0.803
Glucocorticoids, *n* (%)	115 (65.3)	73 (69.5)	24 (49.0)	18 (81.8)	0.010

Abbreviations; ILD: diffuse interstitial lung disease, SAD: systemic autoimmune disease, BMI: body mass index; ACPA: anti-citrullinated peptide antibodies; ANA: antinuclear antibody; UIP: usual interstitial pneumonia, NSIP: nonspecific interstitial pneumonia; f-NSIP: Fibrotic nonspecific interstitial pneumonia (f-NSIP) has been recognized as one of the major types of chronic idiopathic interstitial pneumonia, along with usual interstitial pneumonia/idiopathic pulmonary fibrosis, FVC: forced vital capacity, FEV_1_: forced expiratory volume in the first second, DLCO-SB: diffusing capacity for carbon monoxide (single-breath method), csDMARD: conventional synthetic disease-modifying anti-rheumatic drug, bDMARD: biologic DMARD.

**Table 2:** COVID-19 Infection and Vaccination.

Variable	Total (*n* = 176)	Rheumatoid Arthritis *n* = 105	Systemic Sclerosis *n* = 49	Inflammatory Myopathy *n* = 22	*p*-Value
COVID-19					
COVID-19 infection, *n* (%)	22 (12.5)	14 (13.3)	4 (8.2)	4 (18.2)	0.460
Severe COVID-19 infection, *n* (%)	7 (4.0)	7 (50.0)	0 (0.0)	0 (0.0)	0.085
Died from COVID-19, *n* (%)	3 (1.7)	3 (2.8)	0 (0.0)	0 (0.0)	0.113
Complete COVID-19 vaccination, *n* (%)	163 (92.6)	94 (89.5)	47 (95.9)	22 (100.0)	0.135

Abbreviations; ILD: diffuse interstitial lung disease, SAD: systemic autoimmune disease.

**Table 3:** Multivariate analysis of factors associated with SARS-CoV2 infection in patients with ILD-SAD.

Variable	Univariate OR (95% CI)	Multivariate OR (95% CI)	*p*-Value
Age in years	1.002 (0.967–1.038)		
Male sex	1.092 (0.419–2.845)		
Arterial hypertension	1.787 (0.712–4.484)		
Diabetes mellitus	2.222 (0.660–7.488)		
Obesity (BMI ≥ 30)	2.011 (0.752–5.381)		
FVC% predicted	0.970 (0.943–0.997)	0.971 (0.946–0.989)	0.040
DLCO% predicted	0.984 (0.955–1.000)		
Glucocorticoids (mg/day)	1.035 (0.991–1.080)		
Immunosuppressants	1.500 (0.609–3.695)		
Rituximab	2.408 (1.010–6.507)	3.490 (1.129–6.100)	0.029
Vaccination	0.186 (0.055–0.634)	0.169 (0.030–0.570)	0.004

Naglekerke R2 = 0.243. Abbreviations; ILD: diffuse interstitial lung disease, SAD: systemic autoimmune disease, BMI: body mass index; FVC: forced vital capacity, DLCO: diffusing capacity of the lung for carbon monoxide. Variables included: age, sex, arterial hypertension, diabetes mellitus, obesity, FVC, rituximab, vaccination.

**Table 4:** Multivariate analysis of factors associated with severe SARS-CoV2 infection in patients with ILD-SAD.

Variable	Univariate OR (95% CI)	Multivariate OR (95% CI)	*p*-Value
Age in years	1.012 (0.949–1.079)		
Male sex	1.162 (0.670–1.673)		
Arterial hypertension	1.837 (0.396–8.511)		
Diabetes mellitus	5.761 (2.999–17.660)	4.923 (1.508–19.097)	0.018
Obesity (BMI ≥ 30)	4.708 (1.217–21.777)		
FVC% predicted	0.968 (0.922–1.000)		
DLCO% predicted	0.990 (0.944–1.003)		
Glucocorticoids (mg/day)	1.026 (1.001–1.096)		
Immuno-suppressants	0.673 (0.127–3.572)		
Rituximab	3.621 (1.769–17.049)		
Vaccination	0.020 (0.003–0.119)	0.024 (0.004–0.170)	<0.001

Naglekerke R2 = 0.347. Abbreviations; ILD: diffuse interstitial lung disease, SAD: systemic autoimmune disease, BMI: body mass index; FVC: forced vital capacity, DLCO: diffusing capacity of the lung for carbon monoxide. Variables included: age, sex, arterial hypertension, diabetes mellitus, obesity, FVC, glucocorticoids, rituximab, vaccination.

**Table 5:** Multivariate analysis of factors associated with severe SARS-CoV2 infection in patients with ILD-SAD and SARS-CoV-2 infection.

Variable	Univariate OR (95% CI)	Multivariate OR (95% CI)	*p*-Value
Age in years	1.019 (0.936–1.109)		
Male sex	1.168 (0.660–1.631)		
Type of ILD-SAD	4.083 (0.691–34.920)		
Arterial hypertension	1.125 (0.183–6.935)		
Diabetes mellitus	4.988 (1.390–31.660)		
Obesity (BMI ≥ 30)	5.333 (0.751–37.862)		
FVC% predicted	0.994 (0.937–1.054)		
DLCO% predicted	0.996 (0.949–1.102)		
Glucocorticoids (mg/day)	1.096 (0.904–1.097)		
Immuno-suppressants	0.350 (0.051–2.407)		
Rituximab	3.962 (0.613–13.574)		
Vaccination	0.015 (0.001–0.110)	0.020 (0.002–0.179)	<0.001

Naglekerke R2 = 0.293. Abbreviations; ILD: diffuse interstitial lung disease, SAD: systemic autoimmune disease, BMI: body mass index; FVC: forced vital capacity, DLCO: diffusing capacity of the lung for carbon monoxide. Variables included: age, sex, diabetes mellitus, obesity, FVC, rituximab, vaccination.

## Data Availability

Data presented in this study are available on request from the corresponding author.

## Supplementary Materials

The following supporting information can be downloaded at: https://www.mdpi.com/article/10.3390/jcm11092437/s1, Figure S1: Guideline of vaccination COVID-19 in Andalusia, Spain, 2021; Table S1: Different autoantibodies profiles in different patients with ILD-SAD; Table S2: Factors associated with SARS-CoV-2 infection in 176 patients with ILD-SAD; Table S3: Factors associated with the severity of SARS-CoV-2 infection in 176 patients with ILD-SAD; Table S4: Multivariate analysis of factors associated with severe SARS-CoV2 infection in patients with ILD-SAD and SARS-CoV2 infection.
