# Supplementary material for: Importance of Vaccination against SARS-CoV-2 in Patients with Interstitial Lung Disease Associated with Systemic Autoimmune Disease
Source: J Clin Med. 2022 Apr 26;11(9):2437. doi: 10.3390/jcm11092437 (PMC9106041; doi:10.3390/jcm11092437)
Supplement: Supplementary file 1 [file jcm-11-02437-s001.zip › jcm-1646877-Supplementary Materials.pdf]

Journal of
Clinical Medicine

MDPI

Supplementary Materials

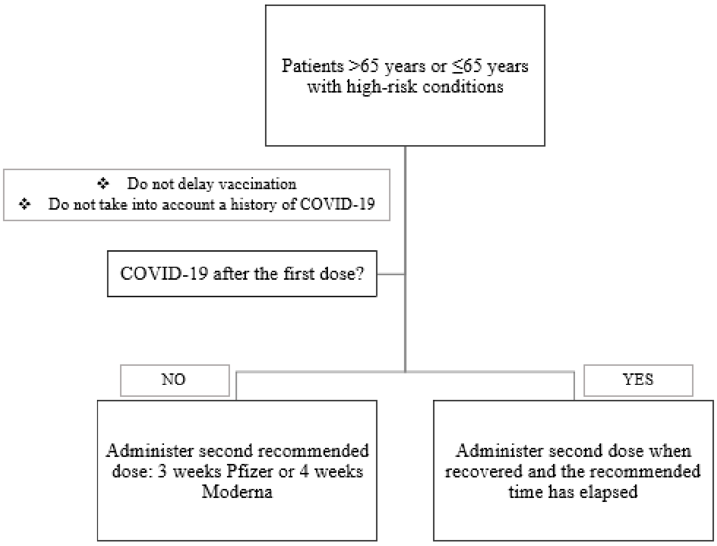

Figure S1. Guideline of vaccination COVID-19 in Andalusia, Spain, 2021.

Table S1. Different autoantibodies profiles in different patients with ILD-SAD.

**Variable**	**Total (n=176)**	**Rheumatoid Arthritis n = 105**	**Systemic Sclerosis n = 49**	**Inflammatory Myopathy n = 22**
Rheumatoid factor, n (%)	104 (59.1)	101 (96.2)	2 (4.1)	1 (4.5)
ACPA (>20), n (%)	91 (51.7)	91 (86.7)	0 (0.0)	0 (0.0)
ANA, n (%)	88 (51.2)	24 (23.8)	46 (93.9)	18 (81.8)
Anti-Scl70, n (%)	30 (17.0)	0 (0.0)	30 (61.2)	0 (0.0)
Anticentromere, n (5)	8 (4.6)	0 (0.0)	8 (16.3)	0 (0.0)
PM-Scl, n (%)	3 (1.7)	0 (0.0)	2 (4.1)	1 (4.5)
Anti-RNP, n (%)	1 (0.6)	0 (0.0)	1 (2.0)	0 (0.0)
Anti-Ku, n (%)	1 (0.6)	0 (0.0)	1 (2.0)	0 (0.0)
RNA-polymerase 3, n (%)	1 (0.6)	0 (0.0)	1 (2.0)	0 (0.0)
Anti-Jo-1, n (%)	8 (4.6)	0 (0.0)	0 (0.0)	8 (36.3)
Anti-PL7, n (%)	5 (2.9)	0 (0.0)	0 (0.0)	5 (22.7)
Anti-EJ, n (%)	1 (0.6)	0 (0.0)	0 (0.0)	1 (4.5)
Anti-NMDA, n (%)	3 (1.7)	0 (0.0)	0 (0.0)	3 (13.6)
Anti-TIF, n (%)	2 (1.2)	0 (0.0)	0 (0.0)	2 (9.1)
Anti-SRP, n (%)	1 (0.6)	0 (0.0)	0 (0.0)	1 (4.5)

Abbreviations; ILD: diffuse interstitial lung disease, SAD: systemic autoimmune disease.

Table S2. Factors associated with SARS-CoV-2 infection in 176 patients with ILD-SAD.

Variable	SARS-CoV-2 Infection n=22	No SARS-CoV-2 Infection n=154	p-Value
Female sex, n (%)	15 (68.2)	102 (66.2)	0.856
Age in years, mean (SD)	64.7 (11.1)	62.4 (12.9)	0.314
Smoking status			0.716
Never smoked, n (%)	14 (63.6)	104 (67.5)	
Smoked, n (%)	8 (36.4)	50 (32.4)	
Arterial hypertension, n (%)	9 (40.9)	43 (27.9)	0.212
Diabetes mellitus, n (%)	4 (18.2)	14 (9.1)	0.188
Obesity (BMI≥30), n (%)	7 (31.8)	29 (18.8)	0.158
Time since diagnosis of SAD, months, median (IQR)	135.4 (67.7-250.2)	130.1 (55.9-216.7)	0.658
Time since diagnosis of ILD, months, median (IQR)	80.9(48.5-114.1)	59.1 (28.5-97.1)	0.041
Radiological pattern			0.752
NSIP, n (%)	10 (45.5)	81 (52.6)	
UIP n (%)	11 (50.0)	64 (41.6)	
Fibrotic NSIP, n (%)	1 (4.5)	9 (5.8)	
Most recent PFT results			
FVC % predicted, mean (SD)	63.9 (18.5)	74.9 (19.8)	0.015
FEV1 % predicted, mean (SD)	65.2 (17.4)	76.8 (18.4)	0.030
DLCO-SB % predicted, mean (SD)	51.9 (14.3)	56.1 (16.5)	0.045
Treatments			
csDMARDs, n (%)	12 (54.5)	99 (64.3)	0.376
Methotrexate, n (%)	5 (22.7)	48 (31.2)	0.419
Leflunomide, n (%)	3 (13.6)	26 (16.9)	0.701
Sulfasalazine, n (%)	2 (9.1)	6 (3.9)	0.274
Hydroxychloroquine, n (%)	3 (13.6)	27 (17.5)	0.649
bDMARDs, n (%)	9 (40.9)	58 (37.7)	0.769
Anti-TNF, n (%)	1 (4.5)	7 (4.5)	1.000
Tocilizumab, n (%)	0 (0.0)	7 (4.5)	0.307
Abatacept, n (%)	2 (9.1)	18 (11.7)	0.720
Rituximab, n (%)	7 (31.8)	25 (16.0)	0.040
Immunosuppressants, n (%)	11 (50.0)	54 (35.0)	0.176
Mycophenolate, n (%)	8 (36.4)	40 (26.0)	0.118
Azathioprine, n (%)	2 (9.1)	12 (7.8)	0.833

Cyclophosphamide, n (%)	1 (4.5)	2 (1.3)	0.271
Antifibrotic, n (%)	1 (4.5)	2 (1.3)	0.271
Glucocorticoids, n (%)	18 (81.8)	97 (63.0)	0.043
Dose of glucocorticoids (mg/day), median (IQR)	5.0 (5.0-10.0)	5.0 (0.0-6.0)	0.034
Vaccinated, n (%)	17 (77.3)	146 (94.8)	0.003

Abbreviations: ILD: diffuse interstitial lung disease, SAD: systemic autoimmune disease, BMI: body mass index; UIP: usual interstitial pneumonia, NSIP: nonspecific interstitial pneumonia, FVC: forced vital capacity, FEV1: forced expiratory volume in the first second, DLCO: diffusing capacity of the lung for carbon monoxide, csDMARD: conventional synthetic disease-modifying antirheumatic drug, bDMARD: biologic DMARD.

Table S3. Factors associated with the severity of SARS-CoV-2 infection in 176 patients with ILD-SAD.

Variable	Severe SARS-CoV-2 Infection n=7	No Severe SARS-CoV-2 Infection n=169	p-Value
Female sex, n (%)	3 (42.9)	114 (67.5)	0.177
Age in years, mean (SD)	66.2 (9.3)	64.9 (12.8)	0.635
Smoking history			0.209
Never smoked, n (%)	4 (57.1)	114 (67.5)	
Smoked, n (%)	3 (42.9)	55 (32.5)	
Arterial hypertension, n (%)	3 (42.9)	49 (29.0)	0.431
Diabetes mellitus, n (%)	4 (57.1)	14 (8.3)	0.001
Obesity (BMI≥30), n (%)	4 (57.1)	32 (18.9)	0.020
Time since diagnosis of SAD, months, median (IQR)	135.4 (67.7-250.2)	130.1 (55.9-216.7)	0.520
Time since diagnosis of ILD, months, median (IQR)	83.0 (33.4-130.1)	59.9 (28.6-88.8)	0.056
Radiological pattern			0.356
NSIP, n (%)	2 (28.6)	89 (52.7)	
UIP n (%)	4 (57.1)	71 (42.0)	
Fibrotic NSIP, n (%)	1 (14.3)	9 (5.3)	
Most recent PFT results			
FVC % predicted, mean (SD)	62.5 (23.4)	75.9 (19.7)	0.022
FEV1 % predicted, mean (SD)	64.9 (12.3)	74.1 (19.7)	0.043
DLCO-SB % predicted, mean (SD)	50.9 (18.1)	55.7 (16.3)	0.049
Treatments			
csDMARDs, n (%)	5 (71.4)	106 (62.7)	0.640
Methotrexate, n (%)	2 (28.6)	51 (30.2)	0.928
Leflunomide, n (%)	2 (28.6)	27 (16.0)	0.379
Sulfasalazine, n (%)	0 (0.0)	8 (4.7)	0.556

Hydroxychloroquine, n (%)	2 (28.6)	28 (16.6)	0.408
bDMARDs, n (%)	4 (57.1)	63 (37.3)	0.289
Anti-TNF agents, n (%)	0 (0.0)	8 (4.7)	0.556
Tocilizumab, n (%)	0 (0.0)	7 (4.1)	0.583
Abatacept, n (%)	2 (28.6)	18 (10.7)	0.143
Rituximab, n (%)	3 (42.9)	29 (17.2)	0.048
Immunosuppressants, n (%)	2 (28.6)	63 (37.3)	0.640
Mycophenolate, n (%)	2 (28.6)	46 (27.2)	0.937
Azathioprine, n (%)	0 (0.0)	14 (8.3)	0.427
Cyclophosphamide, n (%)	0 (0.0)	3 (1.8)	0.722
Antifibrotic, n (%)	1 (14.3)	2 (1.2)	0.115
Glucocorticoids, n (%)	7 (100.0)	108 (63.9)	0.036
Glucocorticoids dose (mg/day), median (IQR)	6.2 (5.0-11.2)	5.0 (0.0-7.5)	0.039
Vaccinated, n (%)	2 (28.6)	161 (95.3)	<0.001

Abbreviations: ILD: diffuse interstitial lung disease, SAD: systemic autoimmune disease, BMI: body mass index; UIP: usual interstitial pneumonia, NSIP: nonspecific interstitial pneumonia, FVC: forced vital capacity, FEV1: forced expiratory volume in the first second, DLCO: diffusing capacity of the lung for carbon monoxide, csDMARD: conventional synthetic disease-modifying antirheumatic drug, bDMARD, biologic DMARD.

Table S4. Multivariate analysis of factors associated with severe SARS-CoV2 infection in patients with ILD-SAD and SARS-CoV2 infection.

**Variable**	**Severe SARS-CoV-2 Infection n = 7**	**No severe SARS-CoV-2 Infection n = 22**	**p-Value**
Female sex, n (%)	3 (42.9)	15 (68.2)	0.107
Age in years, mean (SD)	66.2 (9.3)	64.7 (11.7)	0.677
Smoking history			0.349
Never smoked, n (%)	4 (57.1)	11 (50.0)	
Smoked, n (%)	3 (42.9)	11 (50.0)	
Arterial hypertension, n (%)	3 (42.9)	9 (40.9)	0.899
Diabetes mellitus, n (%)	4 (57.1)	4 (18.2)	0.015
Obesity (BMI≥30), n (%)	4 (57.1)	7 (31.8)	0.107
Type of ILD-SAD			0.060
Rheumatoid arthritis, n (%)	7 (100.0)	14 (63.6)	
Systemic sclerosis, n (%)	0 (0.0)	4 (18.2)	
Inflammatory myopathy, n (%)	0 (0.0)	4 (18.2)	

Time since diagnosis of SAD, months, median (IQR)	135.4 (67.7-250.2)	134.4 (67.7-257.7)	0.859
Time since diagnosis of ILD, months, median (IQR)	83.0 (33.4-130.1)	80.9 (48.5-114-1)	0.880
Radiological pattern			0.647
NSIP, n (%)	2 (28.6)	10 (45.5)	
UIP n (%)	4 (57.1)	11 (50.0)	
Fibrotic NSIP, n (%)	1 (14.3)	1 (4.5)	
Most recent PFT results			
FVC % predicted, mean (SD)	62.5 (23.4)	63.9 (18.5)	0.644
FEV1 % predicted, mean (SD)	64.9 (12.3)	65.2 (17.4)	0.972
DLCO-SB % predicted, mean (SD)	50.9 (18.1)	51.9 (14.3)	0.584
Treatments			
csDMARDs, n (%)	5 (71.4)	12 (54.5)	0.277
Methotrexate, n (%)	2 (28.6)	5 (22.7)	0.655
Leflunomide, n (%)	2 (28.6)	3 (13.6)	0.163
Sulfasalazine, n (%)	0 (0.0)	2 (9.1)	0.311
Hydroxychloroquine, n (%)	2 (28.6)	3 (18.6)	0.163
bDMARDs, n (%)	4 (57.1)	9 (40.9)	0.290
Anti-TNF agents, n (%)	0 (0.0)	0 (0.0)	1.000
Tocilizumab, n (%)	0 (0.0)	0 (0.0)	1.000
Abatacept, n (%)	2 (28.6)	2 (9.1)	0.091
Rituximab, n (%)	3 (42.9)	7 (31.8)	0.448
Immunosuppressants, n (%)	2 (28.6)	10 (45.5)	0.277
Mycophenolate, n (%)	2 (28.6)	6 (27.3)	0.350
Azathioprine, n (%)	0 (0.0)	2 (9.1)	0.311
Cyclophosphamide, n (%)	0 (0.0)	1 (4.5)	0.484
Antifibrotic, n (%)	1 (14.3)	1 (4.5)	0.134
Glucocorticoids, n (%)	7 (100.0)	18 (81.8)	0.187
Glucocorticoids dose (mg/day), median (IQR)	6.2 (5.0-11.2)	5.0 (5.0-10.0)	0.569
Vaccinated, n (%)	2 (28.6)	17 (77.3)	<0.001

Abbreviations: ILD: diffuse interstitial lung disease, SAD: systemic autoimmune disease, BMI: body mass index; UIP: usual interstitial pneumonia, NSIP: nonspecific interstitial pneumonia, FVC: forced vital capacity, FEV1: forced expiratory volume in the first second, DLCO: diffusing capacity of the lung for carbon monoxide, csDMARD: conventional synthetic disease-modifying antirheumatic drug, bDMARD, biologic DMARD.
